# Hendee's Radiation Therapy Physics, the 4th edition

**DOI:** 10.1002/acm2.12550

**Published:** 2019-02-28

**Authors:** Leah Schubert

**Affiliations:** ^1^ Department of Radiation Oncology University of Colorado School of Medicine Aurora CO USA

## 1 OVERVIEW

The 4th edition of Hendee's Radiation Therapy Physics, published by John Wiley & Sons, Inc., is an introductory textbook that provides a broad overview of radiation physics applied to radiation oncology. The textbook covers topics ranging from basic physics principles to fundamental radiation physics of external beam and brachytherapy, and finally underlying principles of technology and techniques commonly used in the clinic.

## 2 GOALS OF THE NEWEST EDITION

The 4th edition is a recently updated version of the original textbook by Bill Hendee, which was originally published in the 1970s and followed by several successful editions. It has been over 10 yrs since the last edition was published, and numerous changes in the radiation therapy field have occurred during that time. Bill Hendee has retired, and new authors, Todd Pawlicki, Daniel Scanderbeg, and George Starkschall, have carried on the textbook's tradition of providing a solid foundation in radiation physics.

The new authors have made specific changes in this new edition. They renamed the textbook to include Hendee's name to honor his significant influence in the field of medical physics (and notably medical physics education). The authors have updated the content of the book to reflect advances in the field over the past 10 yrs. This includes modifying existing chapters, most significantly the imaging chapter to reflect an increased use of digital imaging. The authors have also added new chapters on image‐guided radiotherapy, proton therapy, computer systems and informatics, and patient safety and quality improvement.

The authors have purposefully narrowed the target audience of this book. Noting the difficulty in presenting the level of rigor and details that is appropriate for a very broad audience, the authors have narrowed the audience primarily to radiation oncology residents. The authors’ goal in this new edition is to present the material with the right balance between being accessible to radiation oncology residents but of sufficient depth to provide an understanding of the material at a fundamental level.

The authors have successfully achieved their goals of this edition. Broad overviews of radiation therapy physics principles and clinical applications are nicely presented. The textbook is particularly strong in explaining concepts in order to provide fundamental knowledge, yet in an understandable manner. The content does not go extremely deep in detail and rigor, but rather succinctly presents the information in a clear, yet sufficient way so that the radiation oncology resident can gain understanding of the necessary physics principles and applications in clinical practice.

## 3 ORGANIZATION AND FEATURES

The textbook is approximately 300 pages in length and contains 20 chapters. The chapters are ordered logically to lay a foundation of basic physics, then apply that foundation to current clinical treatments and technologies. I like how this textbook starts with the basics (atomic particles, how they are created, and how they interact with matter), then builds from there in a logical order. A study of how machines generate the particles needed for imaging and treatment is next presented, followed by how those particles coming out of those machines are measured and calibrated, and how that radiation is deposited in patients. The foundation is set to understand the practical aspects of how patients are planned and treated (including external beam and brachytherapy treatments). The textbook provides overviews of additional technologies that are now common primarily imaging and computational topics, and image‐guided radiotherapy and proton therapy. The book ends with various aspects of ensuring the safety of patients and staff, discussing principles of radiation protection, quality assurance, and patient safety and quality improvement.

Each chapter has its own table of contents, a listing of appropriate and successfully met learning objectives, and examples worked out within the text. Each chapter ends with a summary of key points in bullet format, problem sets, and references, with the appendix providing answers to problem sets. The tables are consistently formatted for clarity and visibility. These features help to quickly navigate each chapter and emphasize key points — a need for any busy resident. The majority of the content is presented in standard paragraph format, which is necessary to achieve the goal of providing understanding of the material, and not simply rote memorization. Thus, the chapter formatting and features allow for a nice balance between the two needs of quick access to key information yet adequate explanation of concepts.

## 4 CHAPTER CONTENTS

The chapter contents focus on providing fundamental knowledge of broad applicability. At around 300 pages, the chapter contents cannot go too in depth in any particular topic. However, this is also a strength, particularly for the radiation oncology resident who needs to understand the concepts and extract key knowledge, without digging into numerous details and overwhelming derivations. The content is subsequently not focused on specific technology from vendors or specific variations with different disease sites. While there is updated content describing new techniques and specialty machines (MR‐guided and surface‐guided radiotherapy, cyberknife, and tomotherapy), the content is limited from going into extensive detail.

The textbook particularly excels at explaining difficult concepts in an easy to understand manner. In the first half of the book, the explanations of fundamental radiation physics principles are highly effective. While these concepts may be second‐hand knowledge to physicists who are very familiar with physics on the atomic level, radiation oncology residents may not have been exposed to this topic since undergraduate studies, which can be overwhelming. The last half of the book is updated to include current practices. The book uses figures effectively to explain challenging concepts as well as illustrate important knowledge. The example problems and problem sets are useful in engaging the reader and enhancing the understanding of the material. While the problem sets may be a bit challenging for the radiation oncology resident, they remain at a reasonable difficulty level. For problems requiring calculations, the inclusion of calculation details in the appendix would be helpful in future editions. This may make these problem sets more accessible and better utilized by residents (although working through these problems with a staff physicist would also fulfill that need).

## 5 SUMMARY OF ASSESSMENT

The authors successfully achieve their aim of narrowing the target audience to radiation oncology residents. The order of topics is presented in a logical order. The textbook excels at explaining concepts, while, at the same time, the presentation and features in each chapter provides clear, and fast access to key information. This is not a book made for simple refresher memorization, but focuses on building understanding, which is very important for trainees new to the field. The examples and problem sets allow residents to practice calculations. While they may be challenging for radiation oncology residents, they are at a reasonable level for achieving enhanced understanding of the concept.

I would also highly recommend this textbook to medical physics residents, which can serve as an effective resource to refresh the resident's understanding of important fundamentals, while distilling the more rigorous, detailed graduate coursework into key concepts needed for the clinical work. Medical physics graduate students and dosimetry students could also use this textbook to supplement their training. The broad overview of information and excellent explanations can reinforce concepts and help illuminate basic understanding among the details presented in more rigorous courses. The second half of this edition is focused on clinical applications, which is a nice introduction to those with limited exposure to the radiotherapy clinic.

In summary, the 4th edition of Hendee's Radiation Therapy Physics is a valuable addition to the literature, and it excels in providing understanding, but in an accessible manner. This textbook successfully balances the breadth and depth of material to provide a solid foundation in radiation physics principles. The explanations are succinct, but include the necessary information. This edition is particularly helpful for residents needing a succinct understanding of physics concepts that can be applied to clinical work, but can also serve as a great refresher for medical physics residents and supplement for medical physics and dosimetry students to reinforce understanding and key concepts.

## CONFLICT OF INTEREST

Conflict of interest does not exist.

